# Navigating Care Amid Crisis: The Impact of the COVID-19 Pandemic on Eosinophilic Esophagitis Management in Canada

**DOI:** 10.3390/jcm14196704

**Published:** 2025-09-23

**Authors:** Sunil Samnani, Muhammad Anas Fazal, Krystyna Pokraka, Joel David, Christopher N. Andrews, Michelle Buresi, Dorothy Y. Li, Matthew Woo, Christopher Ma, Milli Gupta

**Affiliations:** 1 Division of Gastroenterology, Faculty of Health Sciences, Department of Medicine, McMaster University, Hamilton, ON L8S 4L8, Canada; 2 Division of Gastroenterology and Hepatology, University of Alberta, Edmonton, AB T6G 2X8, Canada; 3 Division of Gastroenterology, University of Manitoba, Winnipeg, MB R3T 2N2, Canada; 4 Division of Gastroenterology and Hepatology, Department of Medicine, University of Calgary, Calgary, AB T2N 1N4, Canada; 5 Department of Community Health Sciences, University of Calgary, Calgary, AB T2N 1N4, Canada

**Keywords:** COVID, pandemic, foreign body ingestion, food impactions, endoscopy, Eosinophilic Esophagitis, upper gastrointestinal endoscopy, EGD, esophageal biopsies

## Abstract

**Background and Objectives**: The COVID-19 pandemic caused significant disruptions in healthcare services. Foreign body impactions (FBIs), with Eosinophilic Esophagitis (EoE) being one of the leading underlying causes in adults, are some of the most common emergencies and often require endoscopy. The study assesses the impact of COVID-19 on the incidence and outcomes of foreign body impactions (FBIs) requiring endoscopy at Canadian tertiary centres in a single city. **Methods**: Patients presenting to tertiary care hospital emergency departments in Calgary (March 2019–Feb 2022) for FBI were identified using the AACRS (Alberta Ambulatory Care Reporting System) database using International Classification of Disease (ICD-9 and ICD-10) codes (T178, T181) and provincial diagnostic codes (935.1, 530.4) for a foreign body in the esophagus (530.13 and K20.0). One-way ANOVA (SPSS^®^ 27.0) analyzed incidence and disease progression across Pre-COVID-19 and COVID-19 years. **Results**: 759 patients were included in the analysis (274 Pre-COVID-19 (PC: March 2019–Feb 2020), 234 COVID-19 Year 1 (CY1: March 2020–Feb 2021), and 251 COVID-19 Year 2 (CY2: March 2021–Feb 2022)). The mean age remained consistent, with two-thirds being male. Food was the predominant type of FBI (>90%). The incidence of new EoE in EDs declined from PC (60.9%) to CY1 (47.4%) (*p* < 0.001), while endoscopic resolution remained >96%. Follow-up endoscopies in outpatient settings remained stable (~60%). Non-EoE causes of FBI, including esophagitis and cancer, increased in CY2. The mean ED length of stay rose in CY2, but this was not statistically significant (*p* = 0.06). **Conclusions**: This study highlights the resilience of emergent endoscopic care in Calgary during COVID, despite a decline in new EoE diagnoses, which might be due to access barriers.

## 1. Introduction

The coronavirus disease 2019 (COVID-19) pandemic caused a significant impact worldwide [1]. With its appearance in the spring of 2020 in North America, it posed significant challenges in many aspects of human life and exhausted healthcare resources [1,2]. During the initial phase of the pandemic, many elective procedures were postponed or cancelled to accommodate the increased burden of admissions due to viral illnesses [3]. This created downstream effects on healthcare delivery in outpatient, inpatient, and emergency department (ED) settings, as patients were strongly encouraged to stay home. Many studies have shown that admissions for chronic conditions were lower in the initial phase of the COVID-19 pandemic, and that those who were admitted after the initial wave had worse outcomes due to delayed presentation [4,5,6,7,8,9]. Delays in seeking medical attention were due to fear and concerns among patients about contracting the virus [10,11]. Such delays can ultimately increase the morbidity or mortality associated with various conditions and thus result in overwhelming utilization of healthcare resources [7].

Studies looking at admission due to gastrointestinal conditions like cirrhosis showed a decline in admissions from March to April 2020 [12], despite higher sales of pre-disposing risk factors like alcohol during that period [13,14]. Delayed presentation of foreign body ingestion (FBI) cases to ED in pediatric population were more frequent during COVID-19 pandemic [15,16,17], but this has not been evaluated in adults. One study found an increase in the presentation of button battery ingestions in children with significant concerns for morbidity and mortality [17]. FBIs are a relatively common issue and often require endoscopic interventions [16,18]. In adults, such emergencies are often food or bone impaction-related [19,20,21,22], frequently due to Eosinophilic Esophagitis (EoE) [23,24]. Understanding how the COVID-19 pandemic affected access to care for patients across the world, it could have also impacted patients with foreign body impactions and underlying conditions such as EoE. This is clinically important for several reasons. First, delayed presentations of impactions can result in serious complications, including esophageal perforation and increased morbidity, thereby placing additional strain on already limited healthcare resources. Second, EoE is a chronic, relapsing condition that requires both surveillance and timely intervention; interruptions in care may worsen long-term outcomes. Third, examining these patterns provides insight into how healthcare disruptions during a public health crisis disproportionately affect patients with time-sensitive gastrointestinal conditions.

There is limited data on the impact on EoE care and rates of impactions during the pandemic in Canada. Due to the pandemic restrictions affecting access to healthcare, we aim to assess the impact of COVID-19 on the frequency of impactions in adults (>18 years of age) between 2019 and 2022, focusing mainly on patients with EoE. By identifying gaps in access and outcomes during COVID-19, this study can inform preparedness strategies, optimize pathways for urgent endoscopic care, and ultimately improve management for future crises or system disruptions.

## 2. Materials and Methods

### 2.1. Patient Population

This study was conducted as part of a quality improvement initiative, adhering to local research ethics guidelines outlined by ARECCI. The ethics committee was approached for consent to access patient data from 2019 to 2022. The study protocol was approved by the IRB, signed on 5 May 2023, and is available on reasonable request. The authors confirm the study was completed in accordance with the approved protocol, university regulations, and the Declaration of Helsinki.

Data for the project were provided by the Calgary Zone Analytics and Reporting Team. Patients presenting to emergency departments at all tertiary care centres in Calgary, Alberta, between March 2019 and February 2022 with foreign body impaction (FBI) or food bolus obstruction were identified through the Alberta Ambulatory Care Reporting System (AACRS) database, a centralized provincial database. The query utilized International Classification of Disease (ICD-9 and ICD-10) codes (T178, T181) and provincial diagnostic codes (935.1, 530.4, 530.13, and K20.0) specific to esophageal foreign body cases. The study included adult patients aged 18 years and older who presented to any of Calgary’s tertiary care centres and underwent endoscopy for FBI.

We defined cohorts between March 2019 and February 2020 as the Pre-COVID-19 (PC) cohort, because the first case of COVID-19 in Alberta was diagnosed in March 2020 [25,26,27]. Alberta had its second significant peak wave of the COVID-19 pandemic from March to October 2021 [25,26]. Adult patients with FBI between March 2020 and February 2021 were grouped as COVID-19 Year 1 (CY1), and patients between March 2021 and February 2022 were COVID-19 Year 2 (CY2).

### 2.2. Patients Characteristics and Outcomes

Patient charts were manually reviewed by the authors (S.S., M.A.F., K.P., J.D.) to identify those who met the criteria for suspected or confirmed FBI based on initial presentation, imaging findings, or endoscopy (EGD) reports. Demographic information, including age and sex, was collected for each group to identify any shifts in patient population during the pandemic. Clinical characteristics, such as presenting symptoms, medical history, and diagnoses, were documented. Key outcomes, including endoscopic intervention success rates, biopsy performance during initial endoscopy, follow-up endoscopy with biopsies, and length of stay in the emergency department (ED), were evaluated. The follow-up period was 6–12 months. Data on prior treatments for Eosinophilic Esophagitis (EoE) and history of atopic disorders were included to assess differences in treatment patterns over time. Diagnoses such as new EoE cases, esophagitis unrelated to EoE, esophageal stricture, and other conditions were recorded for analysis.

### 2.3. Statistical Analysis

Statistical analysis was conducted using SPSS^®^ version 27.0 for Windows (Armonk, NY, USA). A one-way analysis of variance (ANOVA) with Bonferroni correction was performed to compare differences across the three time periods (PC, CY1, and CY2) in patient demographics, presenting symptoms, and outcomes. Descriptive analysis was used for quantitative variables, including mean and standard deviation or median and interquartile range. Proportions were compared with the chi-square test or Fischer’s exact test as appropriate. Statistical significance was set at *p* < 0.05 for all analyses.

## 3. Results

A total of 759 patient encounters were included in the study (274 in PC group (March 2019–February 2020), 234 in CY1 (March 2020–February 2021), and 251 cases in CY2 (March 2021–February 2022)). The mean age of patients was 52.9 ± 19.44 years in the PC group, 50.1 ± 18.77 years in CY1, and 51.9 ± 20.1 years in CY2 (*p* = 0.218). Gender showed a stable distribution, with males comprising 69%, 70%, and 67%, respectively, of each group (*p* = 0.662) (Table 1).

Food was the predominant type of impaction and symptom presentation in EDs, with 93% of cases (255/274) in the PC group, 98.7% (231/234) in CY1, and 93.6% (235/251) in CY2 (*p* = 0.003). In comparison to symptoms related to food impaction, dysphagia and non-cardiac chest pain were presenting symptoms in <5% of cases. History of foreign body (not food) ingestion was noted in 44/274 (16.1%) cases in the PC group, 27/234 (11.5%) in CY1, and 31/251 (12.4%) in CY2. When comparing between time periods, more patients presented with food impactions in the PC group than CY1 (*p* = 0.016) and fewer patients presented in CY1 than in CY2 (*p* = 0.005).

Patients with a known history of EoE reported impaction in 43 cases during the PC phase, 40 in CY1, and 29 in CY2, with a declining trend noted during the COVID-19 pandemic, which was not statistically significant. Histories of dysphagia, food impactions, EOE, and concomitant type 2 allergic/atopic conditions were not statistically different across the time periods (Table 1). In the PC group, EoE patients with impactions were more likely to not have been on treatments compared to CY2 patients with FBI (*p* = 0.007). Endoscopic management was successful in the majority of cases (>95%) across the groups. Unsuccessful EGDs (23/759, 3%) were primarily due to a large food bolus requiring prolonged sedation and the need for repeat procedure with general anesthesia in the majority of cases. Some patients had sedation-related hemodynamic instability or aspiration requiring repeat intervention, prior esophageal surgeries needing thoracic surgery assistance, a known history of esophageal cancer, and in one case, esophageal perforation due to mucosal friability and edema. Biopsies were performed in 102/274 (37%) cases in the PC group, 83/234 (35%) in CY1, and 81/251 (32%) in CY2 at the time of encounter (*p* = NS). Among these, histologic evidence of active EoE was observed in the majority of cases: 75/102 (~74%) in PC, 50/83 (~60%) in CY1, and 69/81 (~85%) in CY2. The remaining patients showed either nonspecific esophagitis (PC: 20/102 (20%); CY1: 21/83 (25%); CY2: 8/81 (10%) or normal mucosa (PC: 7/102 (6%); CY1: 12/83 (15%); CY2: 4/81 (5%)). Follow-up EGD with biopsies occurred in 162/274 cases (59%) in the PC phase, 142/234 (61%) in CY1, and 150/251 (60%) in CY2 in outpatient settings (*p* = NS). These follow-up EGDs were performed between 3 and 6 months later in cases of no biopsy at the index procedure, or 6–12 months after the start of treatment for EoE in those who received biopsies at the index procedure. Among these patients who underwent follow-up biopsies, histologic remission was observed in 73% patients (205/279). The average length of stay in the ED was 5.87 ± 3.99 h in the PC group, 5.43 ± 3.17 h in CY1, and 6.23 ± 3.76 h in CY2 (*p* = NS).

New EoE diagnoses declined during CY1, with 36 cases compared to 67 PC, followed by an increase to 64 cases in CY2. Other presentations like esophageal strictures unrelated to EoE or non-EoE esophagitis were stable across the groups (Table 2).

## 4. Discussion

This study provides a comprehensive evaluation of the impact of the COVID-19 pandemic on the presentation, diagnosis, and management of patients with FBI in Calgary, Canada. Despite significant disruptions in healthcare due to the pandemic and concerns over COVID-19’s transmission, the proportion of patients receiving an endoscopy or being diagnosed with EoE was not statistically different before and during the pandemic. These results underscore how ED services managed food impactions despite high concerns about healthcare utilization during an unprecedented period.

FBIs are common in the pediatric population, and several studies have shown that numbers trended higher in the early phase of COVID-19 compared to pre-COVID-19 [16,28]. This study found that overall patient numbers accessing the ED decreased, but the number of endoscopic procedures remained largely unaltered by the pandemic, an outcome that was contrary to our initial expectations. The COVID-19 pandemic has significantly impacted healthcare delivery and access. A study assessed the impact of the COVID-19 pandemic in China on patients with ST-elevation myocardial infarction (STEMI) and noted that admissions for STEMI declined, treatment was delayed, and in-hospital mortality rose. Patients experienced longer symptom-to-first medical contact times, reduced rates of primary PCI, greater reliance on fibrinolysis, and higher rates of adverse cardiovascular events, all contributing to poorer overall outcomes [29]. Similarly, a study in the United Kingdom found significant changes in injury patterns, with notable shifts in mechanisms of injury and patient demographics during the pandemic period, with reductions in high-energy accidents and hospital admissions [30]. A particularly striking observation in our study was the decrease in new diagnoses of EoE during CY1, followed by a rebound in CY2. This may be attributed to disruptions in elective services and endoscopic procedures during the early phase of the pandemic. Endoscopies were prioritized despite the restrictions and impactions were treated like emergencies, aligning with studies that showed a temporary decline in elective procedures, which recovered over time [31,32]. Additionally, the rise in cases of esophagitis unrelated to EoE and esophageal cancer in CY2 highlights the evolving clinical profile of patients presenting with impactions over time. Interestingly, despite the overall reduction in patient numbers, our study shows no variability in terms of age, gender, and presentation across pandemic phases. There was variation noted in studies with different demographics, such as pediatric patients accessing EDs more frequently during the pandemic [33].

Notably, more EoE patients were assessed during the pandemic amidst the pandemic’s challenges. This finding reflects how the diagnostic landscape shifted, potentially influenced by the deferment of non-urgent cases and a focus on managing urgent presentations. Although our study did not observe a significant change in the rate of impactions over the pandemic years, the diagnostic yield of EoE remained high. The increase in diagnostic yield of EoE in EDs, as observed in our study, parallels findings from a review that reported improved diagnostic yields despite reduced procedural volumes [34,35]. This increase may be attributed to the focus on fewer, more complex cases and the deferral of non-urgent procedures [36,37].

One of the strengths of this study is its large sample size, which allows for robust comparisons across pre-COVID-19 and pandemic years. The use of a well-established and centralized administrative database (AACRS) ensures comprehensive data capture for the city of Calgary and reduces the risk of selection bias. Additionally, the study employs standardized diagnostic and procedural codes, enhancing the accuracy and reproducibility of the findings. The focus on a specific geographic region (Calgary) within a publicly funded healthcare system provides valuable insights into how healthcare delivery was affected by the pandemic in Canada. Our study also evaluated the length of stay as a marker for severity and hospital utilization during the COVID-19 pandemic and found these patients did not require additional time.

This retrospective observational study is subject to several limitations that may impact the validity and generalizability of its findings. Firstly, the retrospective design introduces inherent risks of information bias due to potential inconsistencies or incompleteness in the administrative data captured within the Alberta Ambulatory Care Reporting System (AACRS). Although the centralized database mitigates selection bias, the analysis did not account for confounding variables, including patient comorbidities, socioeconomic determinants, or barriers to healthcare access, which may influence ED utilization patterns. Potential misclassification of food bolus impaction cases may have occurred due to variability in coding practices, with some cases possibly recorded under broader diagnostic categories such as dysphagia. This could result in an underestimation of true FBI incidence, reflecting challenges in data standardization. The study’s scope was confined to tertiary care EDs within Calgary, potentially excluding patients managed in primary care or community settings, or those who deferred care during the pandemic. This restriction introduces selection bias and limits the external validity of the findings, a concern analogous to the meta-analysis methodology’s exclusion of small observational studies to minimize bias. Another limitation was the absence of patient-reported outcomes, such as symptom severity, functional status, or health-related quality of life, which precludes a comprehensive assessment of the pandemic’s impact on patient well-being. The study’s three-year timeframe, while sufficient to evaluate short-term trends, does not permit analysis of the long-term consequences of the pandemic on ED presentations or management strategies. These limitations necessitate cautious interpretation of the results and highlight the need for prospective, multicentre studies with standardized data collection, adjustment for confounders, and incorporation of patient-centred outcomes to enhance the robustness and applicability of future research.

## 5. Conclusions

In summary, our study provides valuable insights into the effects of the COVID-19 pandemic on GI endoscopy, reflecting and extending the findings of the review literature. Unlike pediatric data, the adult population did not see a drop in patients presenting with impactions. This study contributes to a deeper understanding of how the COVID-19 pandemic affected the management of impactions, particularly in the context of endoscopy access and utilization. Future efforts should focus on addressing gaps in diagnostic and follow-up practices while ensuring equitable access to care, even during healthcare disruptions.

## Figures and Tables

**Table 1:** Baseline characteristics and demographics of patients assessed for foreign body impaction in ER settings before and during COVID-19 pandemic (2019–2022).

Variables	Pre-COVID-19 (Mar 2019–Feb 2020)	COVID-19 Year 1 (Mar 2020–Feb 2021)	COVID-19 Year 2 (Mar 2021–Feb 2022)	*p* Value
Number of Patients	274	234	251	-
Age (mean + SD)	52.9 ± 19.44	50.1 ± 18.77	51.9 ± 20.1	0.218
Sex				0.662
- Male	189 (69%)	165 (70.5%)	168 (66.9%)	
- Female	85 (31%)	69 (29.5%)	83 (33.1%)	
Presenting Symptoms				0.003 *
- Food Impaction	255 (93.1%)	231 (98.7%)	235 (93.6%)	
- Dysphagia	5 (1.8%)	2 (0.9%)	0	
- Chest Pain	2 (0.7%)	0	1 (0.4%)	
- Foreign body	12 (4.4%)	1 (0.4%)	15 (6%)	
Past Medical History				0.239
- History of FBI	44 (16.1%)	27 (11.5%)	31 (12.4%)	
- History of EoE	43 (15.7%)	40 (17.1%)	29 (11.6%)	
- History of Dysphagia	116 (42.3%)	114 (48.7%)	120 (47.8%)	
- None	71 (25.9%)	53 (22.6%)	71 (28.3%)	
Allergy History				0.311
- Yes	71 (25.9%)	53 (22.6%)	51 (20.3%)	
- No	203 (74.1%)	181 (77.4%)	200 (79.7%)	
Prior Treatment for EoE in Patients with Known EoE				0.010 *
- PPI	15 (31.9%)	15 (36.6%)	7 (17.5%)	
- Inhaled Steroids	3 (6.4%)	4 (9.8%)	3 (7.5%)	
- Both PPI and Inhaled Steroids	9 (19.1%)	8 (19.5%)	13 (32.5%)	
- None	16 (34%)	13 (31.7%)	6 (15%)	
Endoscopy Outcomes				0.511
- Successful	264 (96.4%)	229 (97.9%)	243 (96.8%)	
- Not Successful	10 (3.6%)	5 (2.1%)	8 (3.2%)	
Biopsy at Time of Index EGD				0.487
- No	172 (62.8%)	151 (64.5%)	170 (67.7%)	
- Yes	102 (37.2%)	83 (35.5%)	81 (32.3%)	
Follow-Up EGD with Biopsies				0.938
No	112 (40.9%)	92 (39.3%)	101 (40.2%)	
Yes	162 (59.1%)	142 (60.7%)	150 (59.8%)	
Length of Stay in ED (hours ± SD)	5.87 ± 3.99	5.43 ± 3.17	6.23 ± 3.76	0.060

Abbreviations: FBI, foreign body impaction; EoE, Eosinophilic Esophagitis; EGD, esophagogastroduodenoscopy; PPI, proton-pump inhibitor; ED, emergency department; SD, standard deviation. *: Statistically significant *p* value deemed to be <0.05.

**Table 2:** Number of patients with non-EOE etiology of foreign body impaction seen between 2019 and 2022 in ER.

Diagnosis	Pre-COVID-19 (Mar 2019–Feb 2020) *n* = 274	COVID-19 Year 1 (Mar 2020–Feb 2021) *n* = 234	COVID-19 Year 2 (Mar 2021–Feb 2022) *n* = 251
Total EoE	110 (40.1%)	76 (32.5%)	93 (37.1%)
New cases	67 (60.9%)	36 (47.4%)	64 (68.8%)
Esophageal stricture not related to EoE	20 (7.3%)	24 (10.3%)	21 (8.4%)
Schatzki ring	24 (8.8%)	22 (9.4%)	24 (9.6%)
Esophagitis not EoE	19 (6.9%)	15 (6.4%)	35 (13.9%)
Post-surgery/intervention narrowing	7 (2.5%)	4 (1.7%)	1 (0.4%)
Large food bolus	67 (24.5%)	67 (28.6%)	54 (21.5%)
Foreign body (including pill esophagitis)	3 (1.1%)	0	7 (2.8%)
Esophageal cancer	1 (0.4%)	0	5 (2%)
Others (esophageal web/ring, presbyoesophagus, dysmotility, extraesophageal compression, diverticulum)	15 (5.5%)	18 (7.7%)	8 (3.2%)
No food bolus	8 (2.9%)	8 (3.4%)	3 (1.2%)

Abbreviations: EoE, Eosinophilic Esophagitis.

## Data Availability

The data presented in this study are available on request from the corresponding author due to confidentiality reasons.

## Abbreviations

The following abbreviations are used in this manuscript:

COVID	Coronavirus-19
ED	Emergency Department
EGD	Esophagogastroscopy
EoE	Eosinophilic Esophagitis
ER	Emergency Room
FBI	Foreign Body Impaction
PC	Pre-COVID-19
CY1	COVID-19 Year 1
CY2	COVID-19 Year 2
